# A Blind Spectrum Sensing Method Based on Deep Learning

**DOI:** 10.3390/s19102270

**Published:** 2019-05-16

**Authors:** Kai Yang, Zhitao Huang, Xiang Wang, Xueqiong Li

**Affiliations:** State Key Laboratory of Complex Electromagnetic Environment Effects on Electronics and Information System, National University of Defense Technology, Changsha 410073, China; huangzhitao@nudt.edu.cn (Z.H.); christopherwx@163.com (X.W.); beryl_li@163.com (X.L.)

**Keywords:** spectrum sensing, deep learning, convolutional neural networks, long short-term memory

## Abstract

Spectrum sensing is one of the technologies that is used to solve the current problem of low utilization of spectrum resources. However, when the signal-to-noise ratio is low, current spectrum sensing methods cannot well-handle a situation in which the prior information of the licensed user signal is lacking. In this paper, a blind spectrum sensing method based on deep learning is proposed that uses three kinds of neural networks together, namely convolutional neural networks, long short-term memory, and fully connected neural networks. Experiments show that the proposed method has better performance than an energy detector, especially when the signal-to-noise ratio is low. At the same time, this paper also analyzes the effect of different long short-term memory layers on detection performance, and explores why the deep-learning-based detector can achieve better performance.

## 1. Introduction

At present, the spectrum resources that can be allocated have been stretched, while the amount of devices that need to be accessed through the wireless network will increase exponentially with the popularization of new wireless communication technologies, such as the Internet of Things [1]. On the other side, the utilization of spectrum resources is always at a low level. An effective solution to improve the utilization efficiency of spectrum resources and increase the access capacity of wireless networks is to efficiently allocate dynamic spectrum resources, which is the content of intelligent processing research [2,3]. Intelligent processing is designed to enable the system to automatically sense and automatically access the currently available spectrum resources, autonomously learn the current wireless environment, and automatically reconfigure the current system configuration to maximize the utility of the available spectrum resources [4].

The intelligent processing or system intelligence of the communication network includes three aspects: (1) sensing ability; (2) automatic adjustment of the working mode; and (3) learning ability. Perceptual capability is one of the most important features of intelligent wireless networks and enables the perception of the wireless environment. As a key component of cognitive radio, perceptual capability allows wireless devices to adapt to the current working environment and maximize the utilization of current accessible spectrum resources. The perception capability is afforded by spectrum sensing. In a narrow sense, spectrum sensing determines the spectrum availability [4].

From the open literature, the currently available spectrum sensing techniques can be divided into three main categories: matched filter detection, feature-based detection, and energy detection [5,6,7,8,9,10,11,12,13,14,15,16,17,18,19]. A matched filter detector correlates the copy of the licensed user signal with the received signal to maximize the output Signal-to-Noise Ratio (SNR) of the received signal at decision time [5]. For a known signal with additive white Gaussian noise, the signal detection method based on a matched filter possesses the best detection performance. However, all of the information about the licensed user signals is required, and this condition is not always satisfied in practical applications [7]. The most typical signal-feature-based detection method is based on the cyclostationary feature proposed by Gardner, W.A. et al. [15,16,17,18]. Different from background noise, communication signals generally exhibit cyclostationary correlation characteristics, and different signals will exhibit different cyclic correlation characteristics. Based on some known signal parameters, the cyclic spectrum characteristics of signals can be utilized for signal detection. Although the feature-based detection method can obtain excellent detection performance under certain circumstances, a lack of prior information will greatly increase the computational complexity and deteriorate the detection performance. The energy detection method [17] is one of the simplest methods for spectrum sensing. It determines whether the licensed user signal is present or absent by comparing the energy of the observed signal and a predefined threshold. This method does not require any prior information about the licensed user signal, and the computational complexity is low. However, when the SNR is low, the detection performance is ordinary [3,19].

Since prior information on the signal of interest is not always available, especially for non-cooperative communication, a spectrum sensing method that does not require prior information on the signal of interest but possess excellent signal detection performance is desired. The emergence of deep learning [20,21] provides us with the possibility to overcome this difficulty.

**Figure 1:**
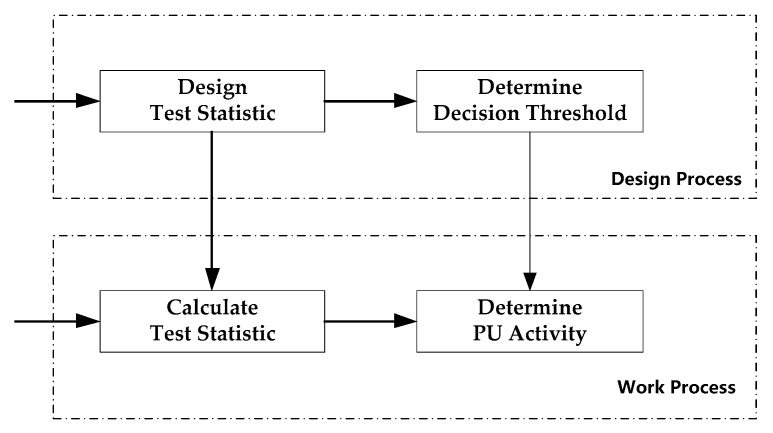
The steps of typical spectrum sensing.

As shown in Figure 1, the steps of typical spectrum sensing methods can be summarized as follows: Firstly, designing the test statistic according to the characteristic difference between the signal of interest (i.e., the Rrimary User (PU) signal) and the noise. Secondly, based on the test statistic and significance level, determining the decision threshold of the presence of the signal of interest. Thirdly, calculating the value of the test statistic based on the received signal. Finally, comparing the decision threshold and the value of the test statistic to determine if the PU signal is present. The first two steps can be considered as the process of designing the spectrum sensing algorithm. The latter two steps can be considered as the process of detecting the PU’s activity. We note that this is very similar to the pattern recognition problem. The design process for test statistics corresponds to the feature design process in pattern recognition, the determination of the decision threshold corresponds to the process of training a classifier in the pattern recognition, the calculation of the test statistic corresponds to the feature extraction process in the pattern recognition, and the decision of the PU’s activity corresponds to the process of classification and identification according to the trained classifier in the pattern recognition. These two problems can be uniformly described as follows: for the input data, firstly designing a function transformation y=F(x) according to the input data x; secondly, determining the classification plane γ according to the y; thirdly, according to the newly arrived data $\tilde{x}$, calculating the corresponding $\tilde{y}$; and, finally, making the decision according to $\tilde{y}$ and the classification plane γ.

As an important method in pattern recognition, machine learning methods have been widely used in the field of spectrum sensing. Reference [22] applies K-Nearest Neighbors (KNN) and Support Vector Machine (SVM) to spectrum sensing. They found that spectrum sensing methods based on KNN and SVM are more adaptive to the changing signal environments than traditional methods. Reference [23] proposed an Artificial Neural Network (ANN) model based on energy detection and cyclic spectrum feature detection to predict the binary channel state. Under the condition of a low SNR, the proposed method can still detect the signals well. By representing the channel status at each time slot as a time series, [24] uses a Multi-layer Feedforward Neural Network (MFNN) model to predict whether the channel of the next time slot is idle based on the previous N slots. Reference [25] introduces the Hidden Markov Model (HMM) and proposes a method to efficiently model the relationship between the current state and multiple past states. Reference [26] constructs a multivariate time series model. Instead of directly modeling the channel state, the model analyzes the cyclic stationary signal characteristics at each time slot, and uses the Recurrent Neural Network (RNN) to predict the evolution of the Radio Frequency (RF) time series data. However, current spectrum learning methods based on machine learning have mostly been applied to the decision process. Limited by the performance of the test statistic, the performance improvement of these algorithms is low.

Deep learning is currently one of the most popular research directions in the machine learning field. Inspired by large-scale ANNs, it is able to adaptively extract more complex and better potential features from input data. It has achieved great success in many fields, such as computer vision, speech recognition, and natural language processing. As a method that can perform pattern recognition tasks well without the need of expert features, deep learning applied to spectrum sensing tasks will be very promising. However, from the open literature, there is still little research in this area. Reference [27] proposes a Stacked-Autoencoder-Based Spectrum Sensing Method (SAESS) and a Stacked-Autoencoder-Based Spectrum Sensing Method with time-frequency domain signals (SAE-TF) to detect the activity states of PU. They are able to detect the PU’s activity solely based on the received signals and without any requirement for prior knowledge of the PU’s signals. The method preliminarily proves the feasibility and effectiveness of the test statistic based on deep learning, but the method is only for the spectrum sensing task of Orthogonal Frequency Division Multiplexing (OFDM) signals.

For the spectrum sensing problem in non-cooperative communication scenarios, this paper introduces a deep learning method into the spectrum sensing field to seek to obtain better signal detection performance even if the SNR is low and prior information on the signal of interest is lacking. Different from the previous methods, the machine learning method is only applied to the decision process. This paper applies the deep learning method to the design process of test statistics, seeks to use the proven excellent feature extraction ability to complete the design of the test statistics (i.e., adaptively learning a function transformation y=F(x) based on the input data), and hopes to improve the performance of blind spectrum sensing from the root.

**Figure 2:**
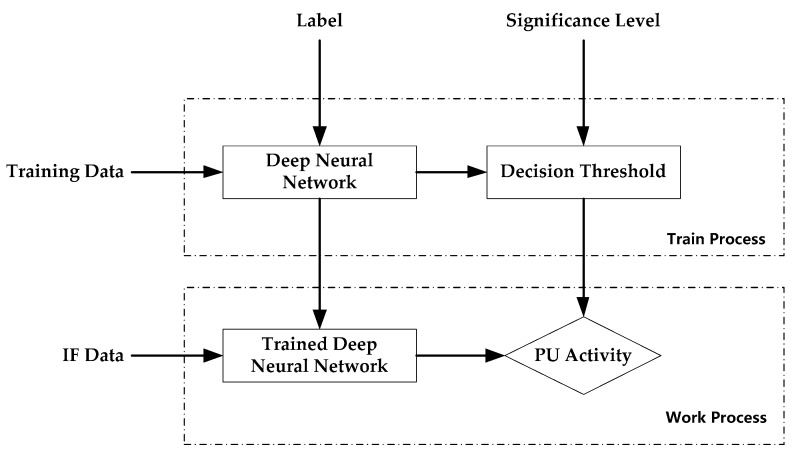
The spectrum sensing method proposed in this paper.

The spectrum sensing method proposed in this paper is shown in Figure 2. Firstly, the deep neural network proposed in this paper is trained using the training data and corresponding labels and saving the optimal deep neural network $NN_{best}$. Secondly, a decision threshold T for determining whether the primary user is present is determined based on the significance level. Thirdly, the new incoming Intermediate Frequency (IF) data is input into the trained deep neural network $NN_{best}$ to obtain its output. Finally, the presence or absence of the primary user is determined based on the output and the decision threshold T. The deep neural network proposed in this paper for spectrum sensing consists of three parts: one-dimensional (1D) convolutional neural networks (1D CNNs), long short-term memory (LSTM), and fully connected neural networks (FCNNs). These three parts constitute the deep neural network and are sequentially connected. The 1D CNN unit can aggregate local features and reduce the dimensions of data through convolutional learning and spatial pooling operations. Therefore, a deep convolutional neural network can extract high-level features and significantly reduce the dimension of the output by alternately using convolution operations and pooling operations multiple times. Raw data are directly used as the input of the deep neural networks in this paper, so the timing of the high-level features of the deep convolutional neural networks output is not broken. Two benefits can be reaped when the input of deep LSTMs is the output of deep convolutional neural networks: (1) deep LSTMs can be used to model the timing patterns of the data; (2) the problem of the high computational complexity inherent in deep LSTMs can be effectively solved and the deep neural networks can be trained more easily since the length of the data processed by deep convolutional neural networks is much lower than that of raw data. The experimental results also show that this deep neural network is suitable for spectrum sensing tasks.

The major contributions of this paper can be summarized as follows:

The design of a deep-learning-based signal detection model using 1D CNNs, LSTM, and FCNNs together. This is an end-to-end signal detection model that does not require any prior information of the signal to be detected, and it can adapt to the signals of most current modulation types. It is inspired by the matched filter and HMM, and can directly use the raw signal as the model input without any hand-crafted feature extraction process.

The model possesses better performance. The experimental results based on the practical communication signals show that the deep-learning-based signal detector proposed in this paper can obtain significantly better detection performance than an energy detector under the condition that prior information on the signal of interest is lacking. Given the condition of an in-band SNR of −9 dB to approximately −5 dB and a false alarm rate of 0.1, the deep-learning-based signal detector can achieve a 25~38% performance improvement over the energy detector.

We analyze the effect of different LSTM layers on detection performance. We find that the optimal signal detection performance can be obtained when the number of LSTM layers is 2.

We explore the mechanism underlying why the deep-learning-based signal detection model can achieve better performance. We find that the trained deep neural network learned some knowledge about the signal of interest, and its filter also behaved like a matched filter.

The remainder of this paper is organized as follows. In the second section, we describe the basis of spectrum sensing theory. The third section describes the system model. The deep neural network’s structure and the related neural network foundation used in this paper are also introduced. The experimental results are in the fourth section. The fifth section summarizes the paper.

## 2. Spectrum Sensing

Perception capability refers to the ability of the system to detect and access parameters that exist in the wireless environment. It is one of the most important features of an intelligent wireless network and a prerequisite for the device to adapt to the environment and maximize the use of accessible spectrum resources. A system’s perception capability is mainly achieved by spectrum sensing. In a narrow sense, spectrum sensing refers to determining whether the spectrum is available at a specific time and location. For a particular frequency band, the goal of spectrum sensing is to decide between the null hypothesis $H_{0}$ and the alternative hypothesis $H_{1}$, where $H_{0}$ indicates that the licensed user signal is absent and $H_{1}$ indicates that the licensed user signal is present. Therefore, spectrum sensing can be expressed as a binary hypothesis test problem:

(1) $$y\left(t\right)=\{\begin{array}{c} w\left(t\right),\\ s\left(t\right)+w\left(t\right), \end{array}\begin{array}{cc} & \begin{array}{c} H_{0}\\ H_{1} \end{array} \end{array},$$

where y(t) indicates the received signal, s(t) indicates the licensed user signal, and w(t) indicates noise or interference.

In spectrum sensing problems, an unlicensed cognitive radio user (also called a second user) is able to utilize the spectrum resources when a licensed user (also called a primary user) is not present or inactive. The performance of spectrum sensing is usually evaluated by the probability of detection and the probability of false alarms. The detection probability is the probability of deciding $H_{1}$ when $H_{1}$ is true; the probability of false alarm is the probability that the decision is $H_{1}$ when $H_{0}$ is true. 

## 3. System Model

**Figure 3:**
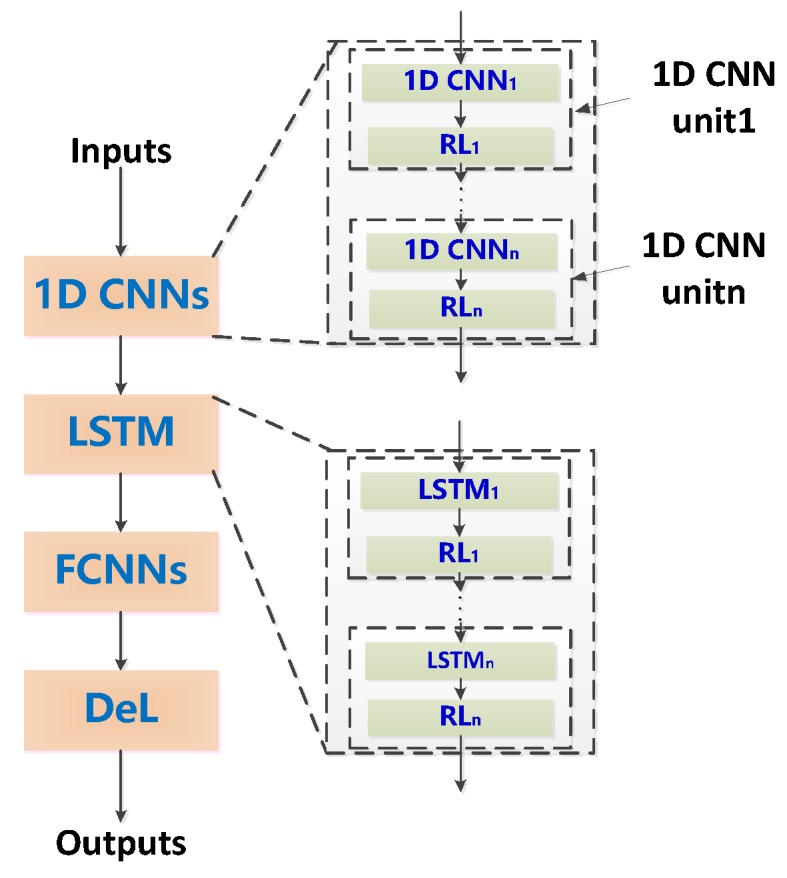
The deep-learning-based detection model proposed in this paper.

In this paper, a deep-learning-based detection model that can be used for spectrum sensing is proposed, as shown in Figure 3. The model consists of three parts: 1D CNNs, LSTMs, and FCNNs.

The 1D CNNs consist of several convolutional units, each consisting of a 1D CNN layer and a regularization layer (RL). The motivation for using 1D CNNs is to expect them to learn to form matched-like filters, extract the signal features from the input data, and obtain filter gains to adapt to the low SNR environment. At the same time, the combination of multiple matched-like filters can improve the robustness of the system. The use of an RL is to speed up training the model and improve the generalization capability of the model.

The LSTMs consist of several LSTM layers and an RL. The motivation for introducing LSTMs is to expect them to establish a more efficient model of the probability distribution of the observed sequence than the HMM, extract the timing features of the signal, and distinguish the signal and noise from the timing regularity of the input data. The role of the RL is the same as above.

The FCNNs use a stack of multi-layer neural networks to form a deep neural network, which refines the output features of the LSTM and attenuates the influence of task-independent features on the decision results. The last layer is the decision layer (DeL) of the entire network, using a linear neural network.

The deep-learning-based signal detection model proposed in this paper directly uses the raw data as the input for the model, and does not need any prior information about the signal of interest. It is an end-to-end blind spectrum sensing method. The following introduces the two neural networks involved in the system model: 1D CNN and LSTM.

### 3.1. 1D CNN

A 1D CNN can be thought of as a variant of a standard neural network. Unlike standard neural networks, where the hidden layer is fully connected, a 1D CNN introduces a special network structure that alternates between the convolution ply and the pooling ply.

#### 3.1.1. Convolution Ply

**Figure 4:**
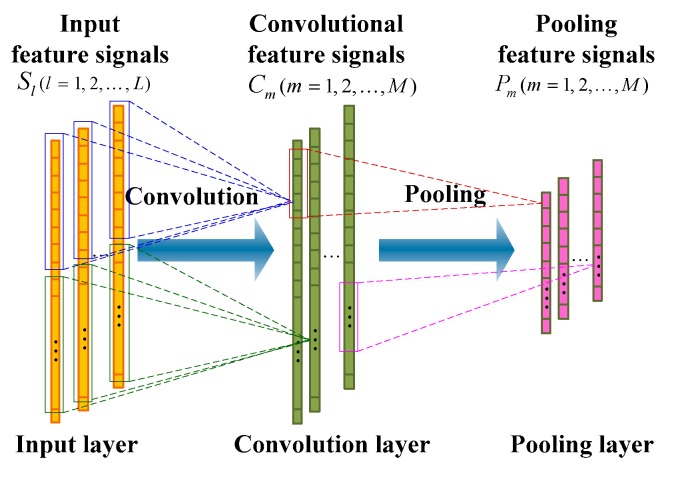
An illustration of one one-dimensional (1D) convolutional neural network (CNN) consisting of a convolution ply and a pooling ply.

As shown in Figure 4, each convolutional feature signal $C_{m}(m=1,2,\ldots ,M)$ is connected to multiple input feature signals through a local weight matrix $W_{m}$ (a matrix of L×F), where F is the length of the convolutional kernel (filter), and determines how many input units each convolutional feature signal unit connects to. The corresponding mapping operation is the so-called convolution in the field of signal processing. That is to say, each unit of the convolutional feature signal can be obtained by the following formula:

(2) $$c_{m,k}=\sigma \left(\sum_{l=1}^{L}\sum_{f=1}^{F}s_{l,f+k-1}w_{l,m,f}+w_{0,m}\right),$$

where σ( ) is the activation function; $c_{m,k}$ is the kth unit of $C_{m}$ of the mth convolutional feature signal; $s_{l,k}$ is the kth unit of $S_{l}$ of the lth input feature signal; $w_{l,m,f}$ is the fth unit of the weight matrix $W_{l,m}$, which connects to the lth feature signal of the input feature signals and maps to the mth feature signal of the convolutional feature signals. Equation (2) can be written in a more compact convolution form:

(3) $$C_{m}=\sigma \left(\sum_{l=1}^{L}S_{l}*W_{l,m}\right)\begin{array}{ccc} & \;(m=1,2,\ldots ,M) & \end{array},$$

where * is the convolution operator, and $S_{l}$ is the lth input feature signal.

There are two differences between the convolution ply and the standard fully connected hidden layer. First, each convolution unit only connects to a portion of the input units. This means that each unit of the convolution layer is a feature extracted by its corresponding partial input units. Second, the units of the convolution layer can be organized into a feature signal, where all units of the feature signal share the same weight vector, but only receive input units from different parts.

#### 3.1.2. Pooling Ply

As shown in Figure 4, the pooling operation occurs after the convolution operation, and a corresponding pooling ply is formed. The pooling ply is also a feature extraction process, which has the same number of feature signals as the convolution ply, except that the feature signal has a lower dimension. The purpose of the pooling operation is to reduce the dimension of the feature signal and enhance the invariance of the feature to deal with small disturbances. The pooling operation applies a pooling function to multiple input feature signal units (convolution feature signal units), wherein the number of input feature signal units of the operation is referred to as the pooling size. Pooling functions usually have a maximum function and an average function. When the maximum pooling function is applied, the pooling layer is defined as:

(4) $$p_{m,k}=\overset{N}{\underset{n=1}{\max}}\left(c_{m,\left(k-1\right)\times q+n}\right),$$

where N is the pooling size; and q is the slide size, which determines the degree of overlap of the adjacent pool windows. Similarly, when applying the average pooling function, the output of the pooling layer is:

(5) $$p_{m,k}=r\sum_{n=1}^{N}\left(c_{m,\left(k-1\right)\times q+n}\right),$$

where r is the scale factor, which can be obtained through training. In image recognition tasks, N=q is usually defined, which indicates that there is no overlap and no gaps in adjacent pooling windows. The reference [28] claims that the performance of maximum pooling is better than that of average pooling. In the work of this paper, we employ the maximum pooling operation.

### 3.2. LSTM

Given the input sequence $s=\left(s_{1},s_{2},\ldots ,s_{T}\right)$, the hidden layer sequence $h=\left(h_{1},h_{2},\ldots ,h_{T}\right)$ and the network output vector $y=\left(y_{1},y_{2},\ldots ,y_{T}\right)$ of the standard RNN are iteratively calculated from t=1 to T by the following formula:

(6) $$h_{t}=H\left(W_{sh}s_{t}+W_{hh}h_{t-1}+b_{h}\right),$$

(7) $$y_{t}=W_{hy}h_{t}+b_{y},$$

where $s_{t}(t=1,2,\ldots ,T)$ is an M-dimensional vector; $h_{t}(t=1,2,\ldots ,T)$ is an N-dimensional vector; $y_{t}(t=1,2,\ldots ,T)$ is a P-dimensional vector; W is a weight matrix (e.g., $W_{sh}$ is an input hidden layer weight matrix); b is an bias vector; and H( ) is an activation function, usually calculated as an element.

**Figure 5:**
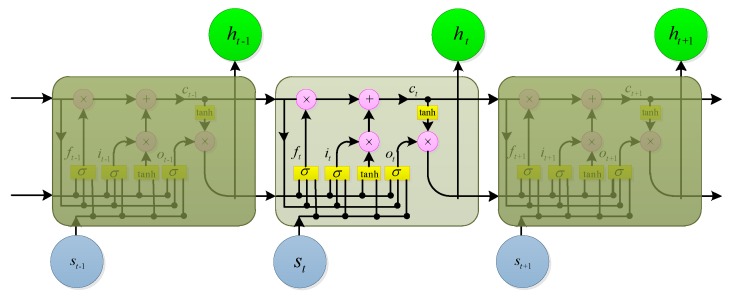
An illustration of a long short-term memory (LSTM) cell.

Usually, LSTMs have better performance than the traditional RNNs. Figure 5 illustrates a simple LSTM cell. In the LSTM version given in [29], H( ) is implemented by the following combination function:

(8) $$i_{t}=\sigma \left(W_{si}s_{t}+W_{hi}h_{t-1}+W_{ci}c_{t-1}+b_{i}\right),$$

(9) $$f_{t}=\sigma \left(W_{sf}s_{t}+W_{hf}h_{t-1}+W_{cf}c_{t-1}+b_{f}\right),$$

(10) $$c_{t}=f_{t}c_{t-1}+i_{t}\tanh\left(W_{sc}s_{t}+W_{hc}h_{t-1}+b_{c}\right),$$

(11) $$o_{t}=\sigma \left(W_{so}s_{t}+W_{ho}h_{t-1}+W_{co}c_{t-1}+b_{o}\right),$$

(12) $$h_{t}=o_{t}\tanh\left(c_{t}\right),$$

where σ is a sigmoid function, i,f,o,c are respectively the input gate, the forgetting gate, the output gate, and the cell activation vector, and their dimensions are consistent with the hidden layer vector h. The weight matrix (e.g., $W_{si}$) from the cell to the gate vector is a diagonal matrix.

An important reason why deep learning can achieve better performance than traditional ANNs is that it has a deeper structure than traditional ANNs, and thus can obtain a higher level of representation of the input data. Deep RNNs can be implemented by stacking hidden layers of multiple RNNs, and the output sequence of the upper layer forms the input sequence of the next layer. Assuming that the same hidden layer function is used for all of the stacked hidden layers, the hidden vector sequence can be iteratively calculated from n=1 to N and from t=1 to T by the following formula:

(13) $$h_{t}^{n}=H\left(W_{h^{n-1}h^{n}}h_{t}^{n-1}+W_{h^{n}h^{n}}h_{t-1}^{n}+b_{h}^{n}\right),$$

where $h^{0}=s$. The output sequence y of the network is calculated by the following formula:

(14) $$y_{t}=W_{h^{N}y}h_{t}^{N}+b_{y}.$$

If LSTM is used for the hidden layer, we can obtain a deep LSTM, which is the structure that will be employed in this paper.

## 4. Experiments

### 4.1. Dataset

**Figure 6:**
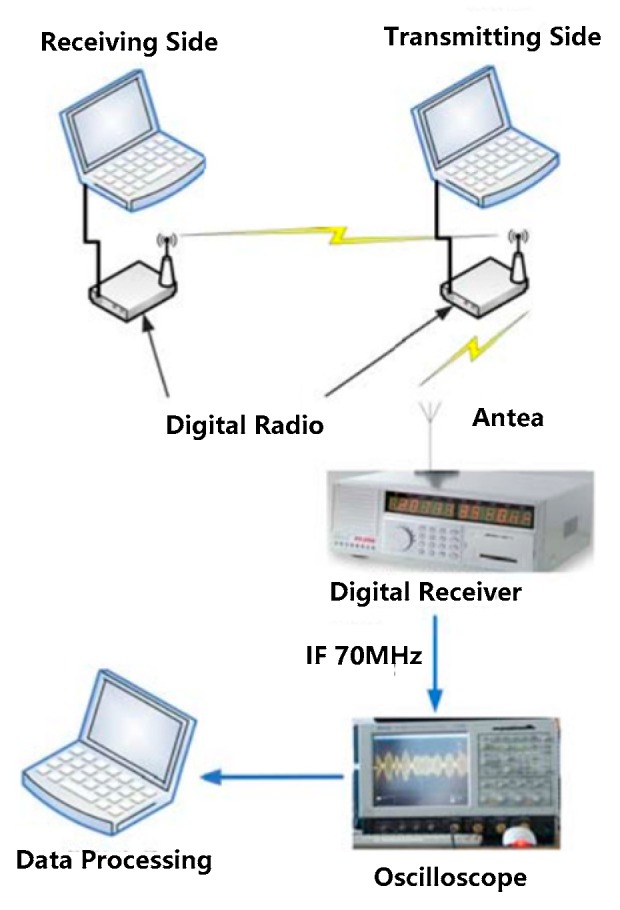
Scheme of the data acquisition system.

The dataset for the experiments in this paper is the RF signal sampled from a digital radio. The experimental data acquisition system is shown in Figure 6. Two wireless digital radios connect to two computers, respectively, which are used as a transmitting side and a receiving side of the system to form a wireless communication link. A digital wireless receiver connected to the horn antenna is used to receive the RF signal from the transmitting side and convert the RF signal into IF 70 MHz. The IF signal is captured by an oscilloscope with a sampling frequency of 250 Msps.

**Figure 7:**
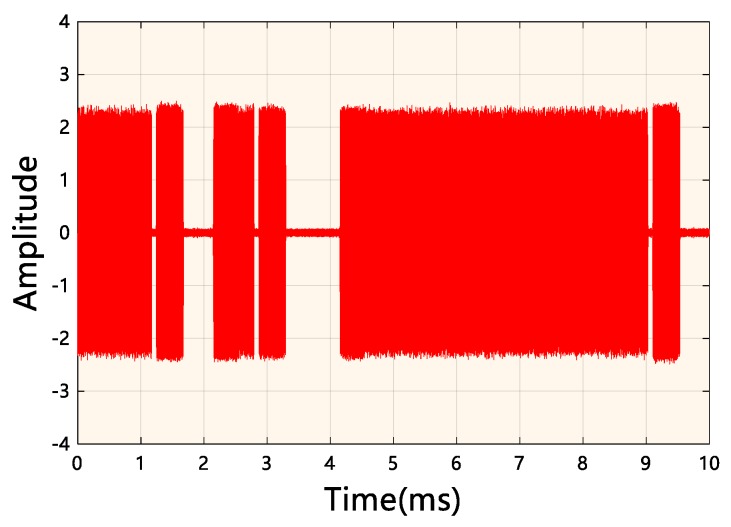
A typical radio signal for the experiment.

The model of the digital radio is AKDS700 (ANYKEY, Wuhan, China). A typical radio signal is shown in Figure 7. The model of the receiver is an SSC024 ultrashort wave digital receiver (Changzhou Radio Factory Co., Ltd., Changzhou, China) with a bandwidth of 20 MHz. The oscilloscope model is a LeCroy Master 8500A (Teledyne LeCroy, New York, NY, USA). The length of collected data is 1 s. Since the distance between the horn antenna and the transmitting side is about 15 cm, this channel can be approximated as an ideal channel, i.e., SNR=∞.

Before the experiment, the data were preprocessed as follows:

1. The data were randomly split into two parts: the training set (50%) and the test set (50%).
2. Each piece of data was filtered. The filter is a rectangular filter with a center frequency of 70 MHz and a bandwidth of 10 MHz.
3. Each piece of data was normalized. Each piece of data had its mean value subtracted from it and was divided by its standard deviation.
4. Noise was generated. First, a white Gaussian noise was generated with the same total length of the training dataset; secondly, the noise was filtered through the same filter as Step 1. Then, the noise was normalized according to Step 2. Finally, the normalized noise was divided into 1-s-long data segments.
5. Noise was added to the training dataset. First, the amplitude of the noise was changed, and the noise amplitude was subjected to a uniform distribution of 0.5–3.5. Then, the noise was added to the training dataset.
6. The training dataset with noise added to it was normalized; the normalization was consistent with Step 2.
7. Each piece of training data was divided into several segments with a length of $L_{0}$. The attributes (noise or signal) of each segment were marked after segmentation.
8. Each piece of test data was divided into several segments with a length of $L_{0}$. The attributes (noise or signal) of each segment’s segmentation were marked.

When the data preprocessing operation had been completed, a training dataset $Train_{0}$ containing 250,000 pieces of segment data and a test dataset $Test_{0}$ containing 250,000 pieces of segment data were obtained, each of which had a length of $L_{0}=1000$. The SNR of the training dataset ranged from −11 dB to 6 dB.

### 4.2. Detection Model Evaluation

In this paper, three experiments were designed to evaluate the proposed model: (1) an evaluation of the detection performance of the deep learning method without prior information on the signal of interest; (2) an evaluation of the effect of different LSTM layers on the detection performance of the deep learning method; and (3) an attempt to explore the mechanism of the excellent detection performance of the deep learning method.

The steps for training and testing the model are as follows:

1. Construct the deep neural network and set the training hyperparameters.
2. Train the deep neural network based on the training dataset $Train_{0}$ and saving the current optimal model $NN_{best}$.
3. Obtain the threshold $T_{SNR=s}$ for determining whether a signal of interest exists under the condition of SNR=s. First, normalize the training dataset and generate noise according to the data preprocessing Steps 2, 3, and 4 in Section 4.1; second, calculate the noise amplitude according to the value of SNR=s; third, change the amplitude of the noise and add it to the training dataset; fourth, normalize the training dataset with added normalized noise; fifth, input the processed dataset into the trained deep neural network $NN_{best}$ to obtain the output; sixth, according to the output of the deep neural network $NN_{best}$, obtain the threshold $T_{SNR=s,i}$ for determining whether the signal of interest exists with a false alarm $P_{f}$; and, finally, run the model 100 times to obtain a threshold $T_{SNR=s}$ for determining whether or not the signal of interest exists under the condition of SNR=s.
4. Obtain the detection probability $P_{d,SNR=s}$ under the condition of SNR=s. First, obtain the test dataset with SNR=s according to Step 3; then, input the test dataset into the trained deep neural network $NN_{best}$ to obtain its output; and, finally, obtain the detection probability $P_{d,SNR=s}$ under the conditions of SNR=s and false alarm probability $P_{f}$ according to the decision threshold $T_{SNR=s}$.
5. Repeating Steps 3 and 4 to obtain the detection probability $\left\{P_{d,SNR=s}\right\}$ with the false alarm probability $P_{f}$ for different SNRs.

All models were constructed, and all training was performed, with the Keras deep learning library using the tensorflow backend. The hardware configuration was as follows: Nvidia 1080Ti graphical processing unit (GPU), Inter Core i7-6800K central processing unit (CPU)@3.4GHz × 12.

#### 4.2.1. Performance Evaluation

The deep-learning-based signal detection method can achieve a high detection probability without prior information on the signal of interest. In order to facilitate an intuitive and a fair comparison of the detection performance of the proposed method, this paper takes an energy detector as a reference since it is a typical detection method that does not need prior information on the signal of interest.

For a completely unknown deterministic signal in a Gaussian white noise channel, according to the generalized likelihood ratio test (GLRT), if there exists

(15) $$T_{ED}=\sum_{i=1}^{L_{0}}y_{i}^{2}>\gamma_{ED},$$

then $H_{1}$ is true for the energy detector, where $L_{0}$ denotes the length of each test data segment. The value of $L_{0}$ is 1000 in this paper. $y_{i}$ indicates the data sample value. When $H_{0}$ is true, there is

(16) $$\frac{T_{ED}}{\sigma^{2}}\sim \chi_{n}{}^{2},$$

where $\sigma^{2}$ represents the noise power; and $\chi_{n}{}^{2}$ is the centralized $\chi^{2}$ distribution of degrees of freedom n. The value of n is $L_{0}$ in this paper. Therefore, the threshold $\gamma_{ED}$ for determining whether the signal of interest exists can be given by

(17) $$\gamma_{ED}=\mathrm{inv}\left(Q_{\chi_{n}{}^{2}}\left(P_{f}\right)\right),$$

where inv( ) represents the inverse of the function; $Q_{\chi_{n}{}^{2}}$ is the right tail probability of $\chi^{2}$; and $P_{f}$ is the false alarm probability. The value of $P_{f}$ is 0.1 in this paper.

The settings of the deep neural network were as follows: five 1D CNN units, two LSTM layers, two FCNN layers, and one DeL. Each 1D CNN unit consists of four parts, which are a convolutional layer, a pooling layer, a normalization layer, and a dropout layer. The parameters of each 1D CNN unit are shown in Table 1. Each pooling layer uses one-dimensional maximum pooling, and pool_size is 2. The normalization layer uses BatchNormalization. The Dropout layer coefficient is 0.4. The number of LSTM cells is 128, the recurrent_dropout size is 0.25, the kernel_regularizer uses L2, and the coefficient is 0.01. The dropout layer was added, followed by the LSTM, and the coefficient is 0.5. Each FCNN layer consists of three parts, followed by a dense layer, a normalization layer, and a dropout layer. Each dense layer consists of 128 units, and the activation function is Rectified Linear Unit (ReLU), the kernel_regularizer uses L1, and the coefficient is 0.001. The normalization layer uses BatchNormalization. The Dropout layer has a coefficient of 0.5. The DeL uses the dense layer, the number of units is 1, and the activation function is a sigmoid function.

The training parameters of the neural network were as follows: the loss function uses binary cross entropy, the optimizer uses Adaptive moment estimation (Adam), the learning rate is 3e − 4, the decay rate is 1e − 8, the batch_size is 64, and the number of epochs is 250. An early-stop strategy was employed.

**Figure 8:**
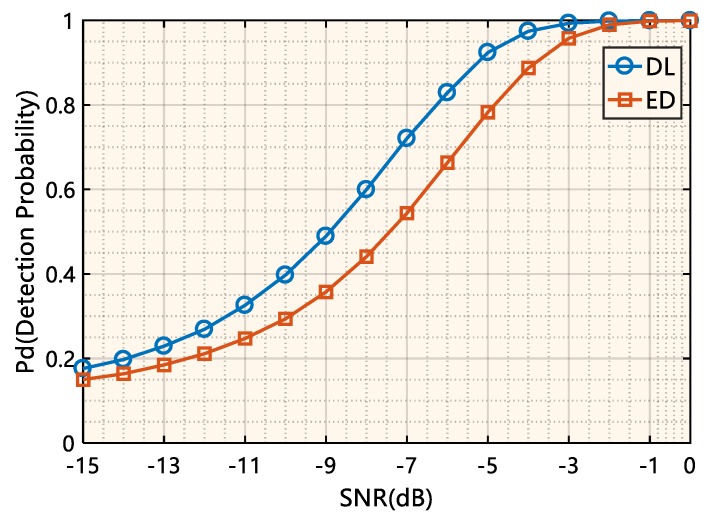
The performance of the deep-learning-based signal detector with a varying signal-to-noise ratio (SNR).

Figure 8 shows the curves of the detection probability $P_{d}$ of the deep-learning-based signal detection method and the energy detection method over a wide range of SNRs with the false alarm probability $P_{f}=0.1$, where ED indicates the energy detection method, and DL indicates the deep-learning-based signal detection method. It can be seen from Figure 8 that the detection performance of the deep-learning-based signal detection method is significantly better than that of the energy detection method regardless of the SNR values. When the in-band SNR is −9 dB to approximately −5 dB, the proposed model can achieve a 25~38% performance improvement over the energy detection method, and this performance improvement does not require the introduction of any prior information on the signal of interest.

**Figure 9:**
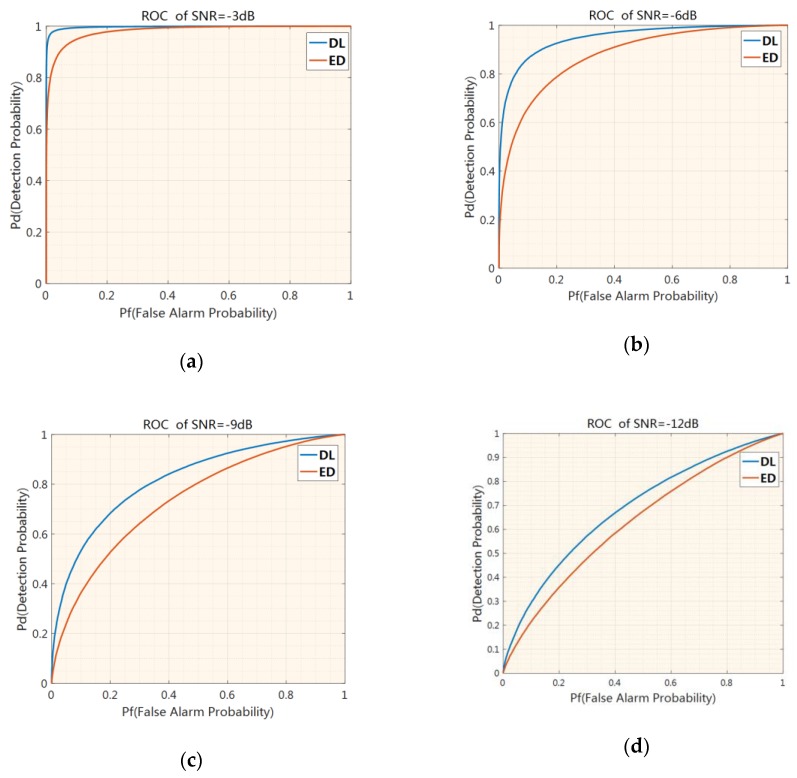
Receiver operating characteristics (ROCs) of the deep-learning-based signal detector versus the energy detector with an SNR of −3 dB (**a**), −6 dB (**b**), −9 dB (**c**), and −12 dB (**d**).

For a more comprehensive comparison, Figure 9 shows receiver operating characteristics (ROCs) of the deep-learning-based detection method and the energy detection method with an SNR of −3 dB, −6 dB, −9 dB, and −12 dB. It can be seen from Figure 9 that the deep-learning-based detection method has obvious performance advantages compared with the energy detection method under the condition of no prior information on the signal of interest.

#### 4.2.2. Effect of Different LSTM Layers

The most obvious difference between an electromagnetic signal and an electromagnetic image is that an electromagnetic signal is one-dimensional data and has a strong temporal correlation. Therefore, the HMM plays a very important role in the field of electromagnetic signal processing. Inspired by this, this paper adds LSTM layers when designing the deep neural networks in order to make the deep neural network have the ability to model the timing law of an electromagnetic signal. This experiment was designed to evaluate the effect of different LSTM layers on the detection performance of the deep learning method.

Table 2 lists the $P_{d}$ of the deep neural network with different LSTM layers at the level of $P_{f}=0.1$ as a function of SNR. The subscript n of ${\mathrm{LSTM}}_{n}$ indicates the number of LSTM layers owned by the deep neural network, and the subscript n=0 indicates that the deep neural network does not use any LSTM layers. The bold number indicates the maximum $P_{d}$ under this SNR. As can be seen from Table 2, deep neural networks using LSTM layer(s) can provide some improvement in detection over deep neural networks that do not use LSTM layer(s). Taken together, the detection performance is optimal when n=2, which can bring about a performance improvement of up to 2% (SNR = −10 dB) compared with n=0. Reference [30] proposed a modulation recognition method solely based on LSTM. The experimental results show that the deep-learning-based method is superior to other standard techniques when the input is raw data. At the same time, consistent with the experimental conclusions of this paper, the best results can be obtained when the number of LSTM layers is 2. We believe that, as mentioned before, this is because a deeper network structure can describe the input signal better. On the other hand, the LSTM with more layers increases the number of parameters that need to be trained, and this makes the training of the model more difficult. The result of this experiment indicates that the LSTMs play a role similar to HMM to a certain extent, and realize the modeling of the temporal correlation of the signals.

#### 4.2.3. Mechanism Exploration

**Figure 10:**
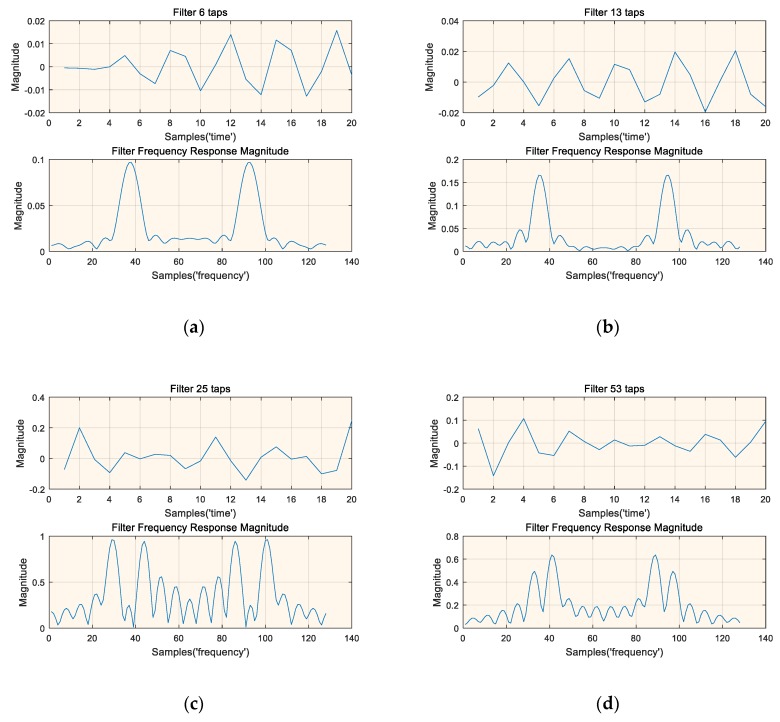
Time and frequency magnitude representations of four typical filters, (**a**) Time and frequency magnitude representations of filter 6 taps, (**b**) Time and frequency magnitude representations of filter 13 taps, (**c**) Time and frequency magnitude representations of filter 25 taps, (**d**) Time and frequency magnitude representations of filter 53 taps.

It is important to explore what each layer of the deep neural network has learned for future work. We have drawn the time and frequency domain magnitude representations of some typical filters, as shown in Figure 10. The frequency response of the filter is the 128-point Fast Fourier Transform (FFT) after zero-padding. The time domain representation of the filter does not show a special meaning, but its frequency response shows strong frequency selectivity. The regularity of the frequency magnitude domain of the filter indicates that the trained deep neural network has learned some knowledge about the signal of interest to some extent. It is important to note that the filters shown in Figure 10 are selected and not all filters can be familiar to an expert. 

**Figure 11:**
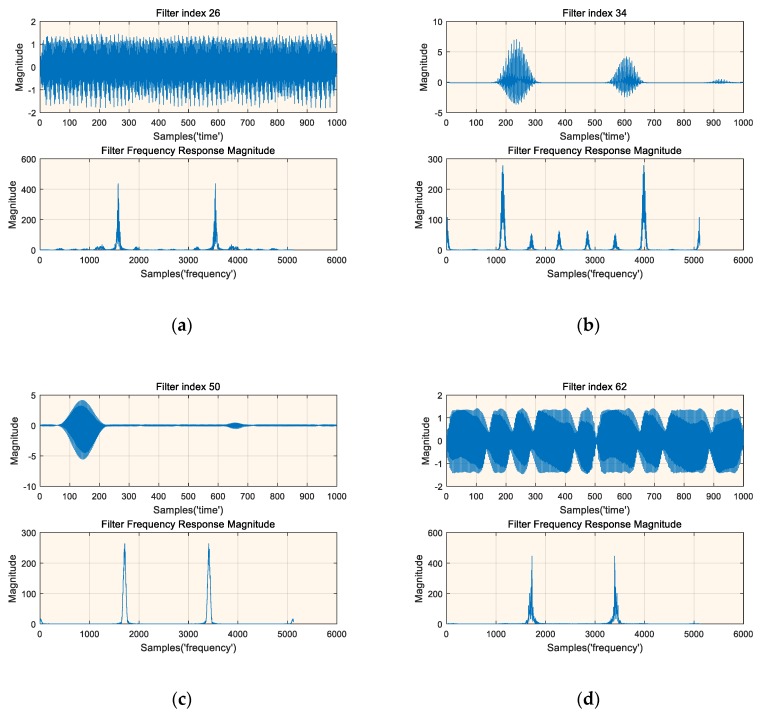
Time and frequency magnitude representations of the signals that most activate the four selected filters, (**a**) Time and frequency magnitude representations of the signals that most activate filter 26 taps, (**b**) ime and frequency magnitude representations of the signals that most activate filter 34 taps, (**c**) ime and frequency magnitude representations of the signals that most activate filter 50 taps, (**d**) ime and frequency magnitude representations of the signals that most activate filter 62 taps.

Another way to visualize these filters is as follows: apply random data as their input, and then perform a gradient descent for the output of a particular filter to find the input data that can most activate the particular convolutional neuron [31]. This can be achieved by the method shown in Equation (18).

(18) $$\begin{array}{ccc} \max{\left\|f\left(x\right)\right\|}_{2} & s.t. & {\left\|x\right\|}_{2}=1 \end{array},$$

where ${\left\|\;\right\|}_{2}$ indicates the l2-norm; and f( ) represents the output of a particular filter. Since the output of the first layer of the neural network has the greatest influence on the subsequent neural network layers and has a high correlation with the input of the neural network, we selected the first layer filter of the neural network for experiment. Figure 11 shows the results of the time domain and the corresponding frequency domain magnitude of the selected four filters, where the frequency response is the 5120 point FFT after zero padding. It can be found that the results of Figure 11a and Figure 11d are very similar to the signal parts of the input data, i.e., the signal activates the filter the most. The results of Figure 11b and Figure 11c also show that the corresponding filter has strong frequency selectivity. This experiment once again shows that the trained deep neural network has learned some knowledge about the signal, and when the signal of interest appears, it can obtain a larger activation output, which is very similar to the mechanism of the matched filter. Therefore, this experimental result also undoubtedly reflects the reason why the deep-learning-based detection method can obtain better detection performance.

## 5. Conclusion

In summary, this paper proposed a spectrum sensing method based on deep learning. Firstly, inspired by the matched filter and HMM, we used a combination of 1D CNN, LSTM, and FCNN to establish a deep-learning-based signal detection model, which is an end-to-end signal detection model that does not need any prior information on the signal to be detected. Next, the experimental results showed that the proposed spectrum sensing method based on deep learning had obvious performance advantages when a priori information on the signal to be detected was lacking. Given the condition of an in-band SNR of −9 dB to approximately −5 dB and a false alarm rate of 0.1, the proposed method can obtain a 25~38% performance improvement compared with the energy detector method. Then, we analyzed the effect of different LSTM layers on the detection performance, and found that the signal detection model proposed in this paper obtained the optimal signal detection performance when the number of LSTM layers was 2. Finally, we explored the mechanism by which deep learning methods can achieve better performance. We found that the trained deep neural network learned some knowledge about the signal, and its filters also behaved like a matched filter.

This paper, as an exploration of the use of deep learning to achieve spectrum sensing, hopes to provide other researchers with a new approach to spectrum sensing. Future work will focus on obtaining better detection performance with less computing resources.

## Figures and Tables

**Table 1:** Parameters of 1D CNNs.

CFENs	Filters	Kernel_Size	Padding	Activation	Kernel_Regularizer
1D CNN unit 1	64	20	same	ReLU	L2(0.0001)
1D CNN unit 2	128	20	same	ReLU	L2(0.0001)
1D CNN unit 3	256	10	same	ReLU	L2(0.0001)
1D CNN unit 4	256	10	same	ReLU	L2(0.0001)
1D CNN unit 5	512	5	same	ReLU	L2(0.0001)

**Table 2:** Performance of different LSTM layers with a varying SNR.

	−12 dB	−11 dB	−10 dB	−9 dB	−8 dB	−7 dB	−6 dB	−5 dB	−4 dB	−3 dB
${\mathrm{LSTM}}_{0}$	0.2941	0.3534	0.4271	0.5297	0.6367	0.7521	0.8509	0.9292	0.9721	0.9929
${\mathrm{LSTM}}_{1}$	0.2965	0.3527	0.4497	0.5315	0.6367	0.7487	0.8571	0.9324	0.9737	0.9932
${\mathrm{LSTM}}_{2}$	0.3045	0.3496	**0.4532**	**0.5448**	0.6432	**0.7597**	**0.8661**	**0.9373**	**0.9789**	**0.9946**
${\mathrm{LSTM}}_{3}$	**0.3080**	**0.3652**	0.4364	0.5395	**0.6490**	0.7497	0.8539	0.9332	0.9759	0.9924
